# Chemometrics Methods for Specificity, Authenticity and Traceability Analysis of Olive Oils: Principles, Classifications and Applications

**DOI:** 10.3390/foods5040077

**Published:** 2016-11-17

**Authors:** Habib Messai, Muhammad Farman, Abir Sarraj-Laabidi, Asma Hammami-Semmar, Nabil Semmar

**Affiliations:** 1 Laboratory of Biomedical Genomics and Oncogenetics, Institut Pasteur de Tunis, University of Tunis El Manar, 1002 Tunis, Tunisia; habib.messai@gmail.com; 2 Department of Chemistry, Quaid-i-Azam University, 45320 Islamabad, Pakistan; farmanpk@yahoo.com; 3 Laboratory of Bioinformatics, Biomathematics and Biostatistics (BIMS), Institut Pasteur de Tunis, University of Tunis El Manar, 1002 Tunis, Tunisia; sarraj.abir@live.fr; 4 National Institute of Applied Sciences and Technology (INSAT), University of Carthage, 1080 Tunis, Tunisia; asma.hamami@gmail.com; 5 Laboratoire de Biomathématiques, Faculté des Sciences de Saint-Jérôme, Aix-Marseille Université, 13397 Marseilles, France

**Keywords:** chemometrical methods, olive field, blends, quality control, ordination, clustering, pattern recognition, prediction, chromatographic profiles, spectral data

## Abstract

Background. Olive oils (OOs) show high chemical variability due to several factors of genetic, environmental and anthropic types. Genetic and environmental factors are responsible for natural compositions and polymorphic diversification resulting in different varietal patterns and phenotypes. Anthropic factors, however, are at the origin of different blends’ preparation leading to normative, labelled or adulterated commercial products. Control of complex OO samples requires their (i) characterization by specific markers; (ii) authentication by fingerprint patterns; and (iii) monitoring by traceability analysis. Methods. These quality control and management aims require the use of several multivariate statistical tools: specificity highlighting requires ordination methods; authentication checking calls for classification and pattern recognition methods; traceability analysis implies the use of network-based approaches able to separate or extract mixed information and memorized signals from complex matrices. Results. This chapter presents a review of different chemometrics methods applied for the control of OO variability from metabolic and physical-chemical measured characteristics. The different chemometrics methods are illustrated by different study cases on monovarietal and blended OO originated from different countries. Conclusion. Chemometrics tools offer multiple ways for quantitative evaluations and qualitative control of complex chemical variability of OO in relation to several intrinsic and extrinsic factors.

## 1. Introduction

**Figure 1:**
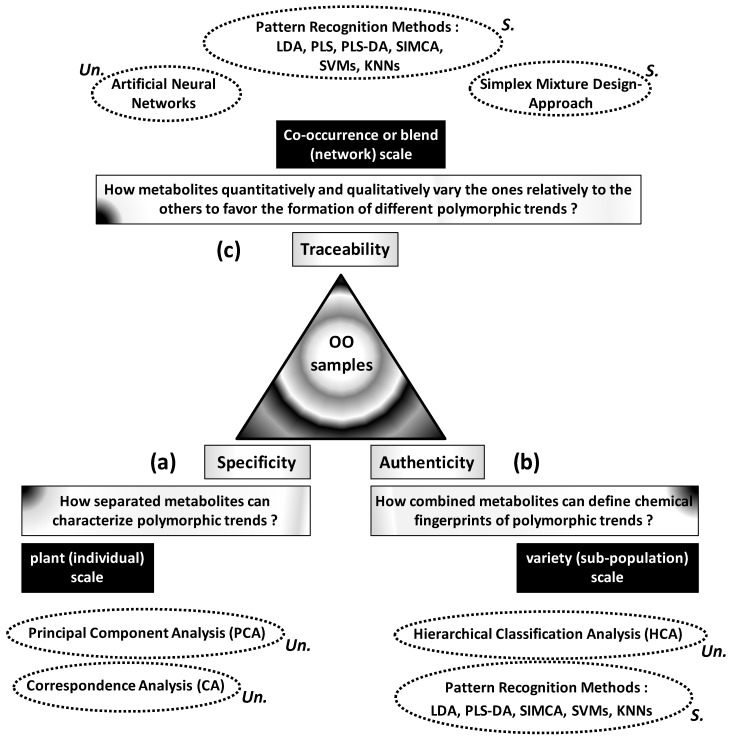
General interests of applications of different statistical methods for specificity (**a**), authenticity (**b**) and traceability (**c**) analysis of olive oil (OO) samples. Some methods are unsupervised (Un) whereas others are supervised (S).

Olive oils (OOs) are complex food matrices due to their highly variable compositions. Different OO cultivars are associated with different environmental living conditions, and are characterized by different chemical patterns resulting in different organoleptic properties [1,2,3]. Thus, chemical patterns play central interests between influencing environment and produced quality of olive products. These interests related to intrinsic regulation and extrinsic sensitivity of metabolic profiles helping for specificity, authenticity and traceability analysis of OO samples and populations [4,5,6,7,8]:

Specificity, authenticity and traceability of complex OO samples can be more or less controlled through statistical analysis of variability between metabolic profiles. Links between these three basic concepts and metabolic variability can be organized around three questions (Figure 1):

- What are the roles and status of separated metabolites in the polymorphism of OO samples (Figure 1a)?
- What is the usefulness of metabolic profiles or spectroscopic features for authentication of OO samples (Figure 1b)?
- How and how much the metabolites’ levels vary the ones relatively to the others to favour the development or formation of well-distinct OO patterns? (Figure 1c)

The first question focuses on highlighting of high or low regulation levels of some metabolites specifically to some phenotypes, cultivars or environmental conditions. Metabolite-phenotype specificity can be statistically highlighted by ordination methods including principal component analysis (PCA) and correspondence analysis (CA).

The second question refers to authenticity consisting in combining several metabolites to define chemical fingerprints from which different OO samples can be reliably classified or predicted. Classification of a wide set of OO samples into different authentic groups is statistically carried out by hierarchical cluster analysis (HCA). However, affiliation of outside samples into appropriate groups implies predictive models based on pattern recognition techniques including linear discriminant analysis (LDA), Soft Independent Modelling of Class Analogies (SIMCA), Support Vector Machines (SVMs) and K-Nearest Neighbours (KNNs). Beyond this qualitative aspect, OO blends can be quantitatively evaluated by predicting some target characteristics or components from high number of input variables (e.g., spectroscopic data). This question can be treated by partial least square (PLS) regression. 

The third question implies the analysis of memorized and interactive variations within heterogeneous matrices. This traceability question requires statistical techniques able to absorb and separate different types of variations associated with (or at the origin of) different polymorphic or multi-aspect patterns. These techniques include artificial neural networks (ANNs) and simplex mixture design-based approach.

Apart from the goal-based criterion, these different statistical methods can be classified into supervised and not-supervised types. The first ones do not need any preliminary information and provide neutral (not guided) results which will help to understand complex structures of studied systems. These techniques include PCA, CA and HCA. However, supervised methods are guided by preliminary information which will serve as reference for target final results. Supervised techniques include LDA, PLS, SIMCA, SVMs, KNN and simplex approach. Finally, ANNs, represent a set of methods including supervised or unsupervised ones.

This review provides an illustrated presentation of traditional and modern chemometrics methods applied in olive oil field. Its ultimate objective is to provide a guideline linking directive questions of authenticity, specificity and traceability to appropriate chemometrics methods. Basic methodological aspects are completed by several recent illustrative applications showing the wide interests and perspectives of chemometrics in this food field. Applications essentially concerned chromatographic and spectroscopic data including HPLC, GC, UV, NIR, MIR, NMR data, etc. in addition to genetic markers. Chemical data concerned several types of metabolites including fatty acids (FAs), triacylglycerols (TGCs), sterols, phenols, volatiles, etc. 

Crucial interests of chemometrics analysis of olive oil samples included geographical and varietal origins determination, standard composition control (conformity, purity, adulteration), historic handling process detection, proportions’ evaluations of mixed OO cultivars in binary and multivarietal blends.

These qualitative pattern recognitions and quantitative evaluations of compositions usefully help for more precise quality control and adulteration reduction.

## 2. Chemometrics Analysis of Specificity 

Specificity refers to the research of several elementary traits which are specific or characteristic of OO sample leading to its distinction within a polymorphic complex system. Several OO samples associated with different geographical origins/environmental conditions were characterized by different variation ranges and trends of metabolites. Specificity of different samples can be highlighted by ordination methods including principal component analysis (PCA) and correspondence analysis (CA).

### 2.1. Principal Component Analysis and Correspondence Analysis 

#### 2.1.1. General Principle

PCA and CA are multivariate methods helping to graphically visualize trends of individuals governed by partial correlations between variables characterizing a polymorphic system [9,10,11,12,13]. This is carried out by compression of the large-dimension and highly dispersed initial dataset into a small and well-structured space. This structuration is provided by new components, called principal components (CPs), combining all the initial variables. PCs represent orthogonal directions along which the total variation is decomposed into structured blocks; such blocks show hierarchically decreasing coverage of total variation.

PCA and CA are carried out by analysing variability of both columns (variables) and rows (individuals) leading to dual analysis which highlights partial links between variables and subsequent behaviours of individuals. By this way, individuals with similar or opposite behaviours are topologically identified by particular levels and trends between some variables. This leads to identify specificity of several groups of individuals on the basis of high or low levels for some variables (positive or negative trends between them).

#### 2.1.2. Methodological Steps

**Figure 2:**
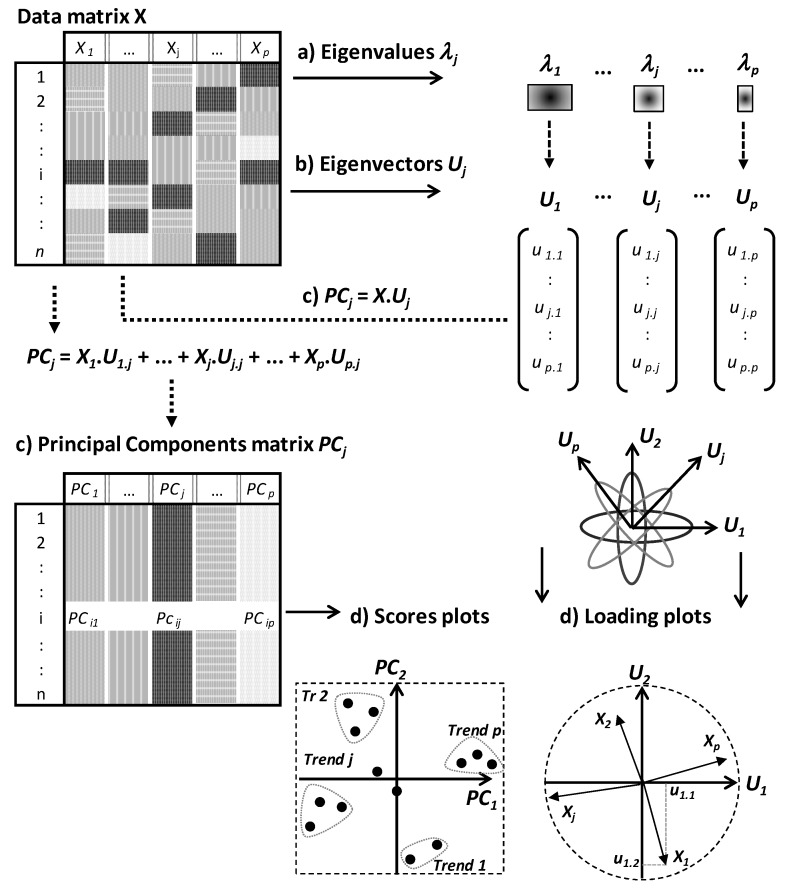
Different methodological (computational and visualization) steps (**a**–**c** and **d**, respectively) of principal components analysis and correspondence analysis.

PCA and CA algorithms can be summarized by three steps consisting of eigenvalues, eigenvectors and PCs calculations (Figure 2).

The number of PCs is equal to that of eigenvalues and eigenvectors, and is defined by the lowest dimension of data matrix X. If *p* < *n*, one expects to calculate p eigenvalues *λ_j_*, *p* associated eigenvectors *U_j_* followed by *p* PCs (*PC_j_*).

Eigenvalues *λ_j_* are weighting values quantifying the relative part of total variation along different PCs (*PC_j_*) (Figure 2a). Eigenvectors are orthogonal and define the spatial directions of PCs (Figure 2b,d). PCs contain the new coordinates of individuals and are calculated by linear combinations between the eigenvectors and initial data matrix *X* (Figure 2c). In the *p* linear combinations, the coordinates of eigenvectors represent algebraic coefficients quantifying the roles of different variables to the *p* PCs. 

Finally, the two topological spaces (called score and loading plots) are considered in parallel ways to analyse the relative behaviours of individuals under the effect of partial correlations between variables (Figure 2d). 

From the subspace defined by a given factorial plot *P_j_P_k_*, different trends are identified from the projection of different points at the extremities of the different PCs. Spatial proximity between projected points means generally similar behaviours toward the different variables. On the other hand, in the factorial plot of variables, positive, negative or not significant correlations are indicated by close, opposite or orthogonal projection of points, respectively (Figure 2d). For example, Figure 2d shows partial negative correlations between *X*_1_ and *X*_2_ along *PC*_2_ and between *X_j_* and *X_p_* along *PC*_1_.

Finally, by considering both row and column plots, some individuals are specifically characterized by some variables on the basis of their projection in a same subspace. A variable has a particularly high or low level in an individual if the two points (individual point and variable point) are projected in a same subspace or two opposite subspaces, respectively. These graphical analyses help to identify particular subpopulations that can be indicative of different phenotype or polymorphic trends. For instance, Figure 2d shows four trends (*Tr*) among which, *Tr1* and *Tr2* are characterized by high levels of variables *X*_1_ and *X*_2_, respectively. Moreover, these trends are opposite along the second principal component *CP*_2_ because of negative correlation between *X*_1_ and *X*_2_.

#### 2.1.3. Application of PCA and CA in OO Field 

In OO field, PCA and CA were used to highlight the best regulated metabolites or characteristic metabolic profiles of different biological varieties leading to a better distinction between them.

**Figure 3:**
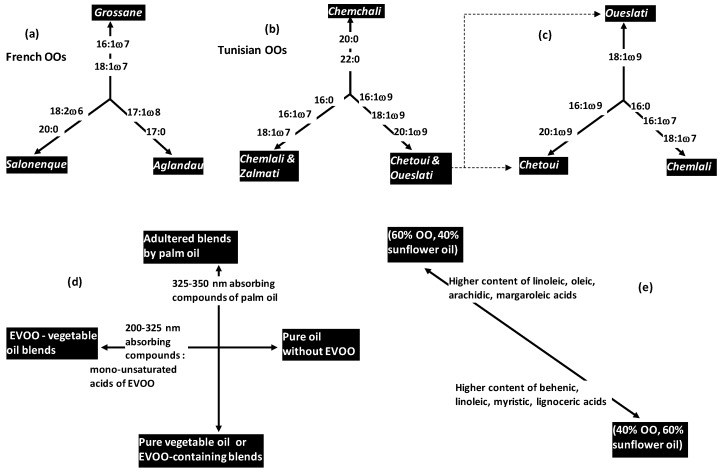
Different study cases (**a**–**e**) on specificity of different OO monocultivars (**a**–**c**) or OO blends (**d**,**e**) by ordination analysis (principal component analysis (PCA) or correspondence analysis (CA)) applied on different physical-chemical parameters (fatty acids, UV absorbances).

CA was applied to extract typical metabolic profiles of three French virgin olive oils (VOOs) characterized by fatty acid profiles: *Aglandau*, *Grossane* and *Salonenque* [14]. The three cultivars showed relatively higher regulations toward some fatty acids (FAs): 17:0 and 17:1ω8 for *A*, 16:1ω7 and 18:1ω7 for *G*, 18:2ω6 for *S* (Figure 3a).

Among ordination analyses, PCA was applied for inter-countries varietal differentiation from FAs and squalene chromatographic profiles containing major and minor compounds [15]. PCA helped to differentiate:

- Between Tunisian (6 varieties), Algerian (6 varieties) and Moroccan VOOs (1 variety) on one hand,
- Between different Tunisian VOOs on other hand.

Tunisian and Moroccan VOOs showed clear chemical differentiations compatible with high geographical distance between the two countries. Also, clear differentiations were highlighted between Algerian and Tunisian cultivars expect for the Blanquette variety which showed some overlapping with Tunisian *Chetoui* variety. 

The same PCA-based work showed different trends of different VOO varieties originated from a same country (Tunisia) (Figure 3b):

- *Chemchali* occupied specific topological subspace due to relatively high level of saturated FAs (20:0, 22:0).
- *Chemlali* and *Zalmati* showed relatively high level of three upstream chained metabolites: 16:0 → 16:1ω7 → 18:1ω7. These two varieties were characterized by C7-monoiunsaturattion vs. C9-monounsaturation in *Chetoui* and *Oueslati*.
- These two later varieties (*Chetoui* and *Oueslati*) were characterized by relatively high levels of C9-monounsaturated FAs (16:1ω9, 18:1ω9, 20:1ω9) linked to sequential metabolic elongation processes.

In another study, Semmar et al. (2016) [16] highlighted chemical differentiation between *Chetoui* and *Oueslati* by applying correspondence analysis (CA) on FAs (Figure 3c).

Although these two varieties showed relatively higher regulation of C9-monounsaturated FAs compared to *Chemlali*, *Chetoui* had more affinity for 16:1ω9 and 20:1ω9 than *Oueslati* which showed relatively higher regulation of 18:1ω9. Moreover, CA highlighted the lowest 16:1ω7 and 18:1ω7 levels in *Chetoui*, two minor FAs characterizing *Chemlali* (because of maximal regulations). However, *Oueslati* showed the lowest level for 18:2ω6 occurring at significantly higher level in *Chetoui* and *Chemlali*.

At higher complexity level, PCA was applied to characterize several blends combining VOO with other vegetable oils. This differential characterization is needed to avoid or reduce adulteration of food oils: (1) some oils are often used as adulterant in OO blends because they are relatively cheap; (2) virgin olive oil (VOO) can be added in other vegetable oils for nutritional values and economic reasons [17,18].

In this field, PCA was applied on UV-spectrometry data to highlight different variation poles specific to different extra virgin olive oils (EVOOs) showing different adulteration types and levels due to presence-absence of corn oil, palm oil, soybean oil and sunflower oil (Figure 3d) [4]. PCA applied on a dataset of mono-, bi- and tri-oils samples revealed that bi-component oil blends containing EVOO were clearly separated from mono-component (pure) oils along the first principal components. This good separation was revealed to be due to high content of EVOO-containing blends in terms of compounds absorbing at 200–325 nm. The authors suggested that compounds could be mono-unsaturated FAs (mainly oleic acid) which are abundant in EVOO [4,19]. The second PC separated pure and EVOO-containing blends from adulterated blends by palm oil (PO). Loading plot of UV-variables showed that this separation was specifically due to PO-characterizing compounds absorbing at 325–350 nm.

PCA was applied to highlight association between quantitative levels of FAs and binary blends mixing OO with sunflower oil at the proportions of 50%–50%, 60%–40% and 40%–60% (Figure 3e) [18]. Blends containing 60% OO were characterized by relatively high concentrations of linolenic, oleic, arachidic and margaroleic acids. This was explained by the fact that these FAs showed higher content in OO than sunflower oil. However, blends containing 40% OO were characterized by other FAs including behenic, linoleic, myristic and lignoceric acids which are more concentrated in sunflower oil.

## 3. Chemometric Analysis of Authenticity 

Authenticity refers to the definition of general patterns of samples integrating different specific characteristics. In OO filed, this helps to classify varieties and to confirm identities of denominated samples.

### 3.1. Hierarchical Cluster Analysis (HCA) 

#### 3.1.1. General Principle

**Figure 4:**
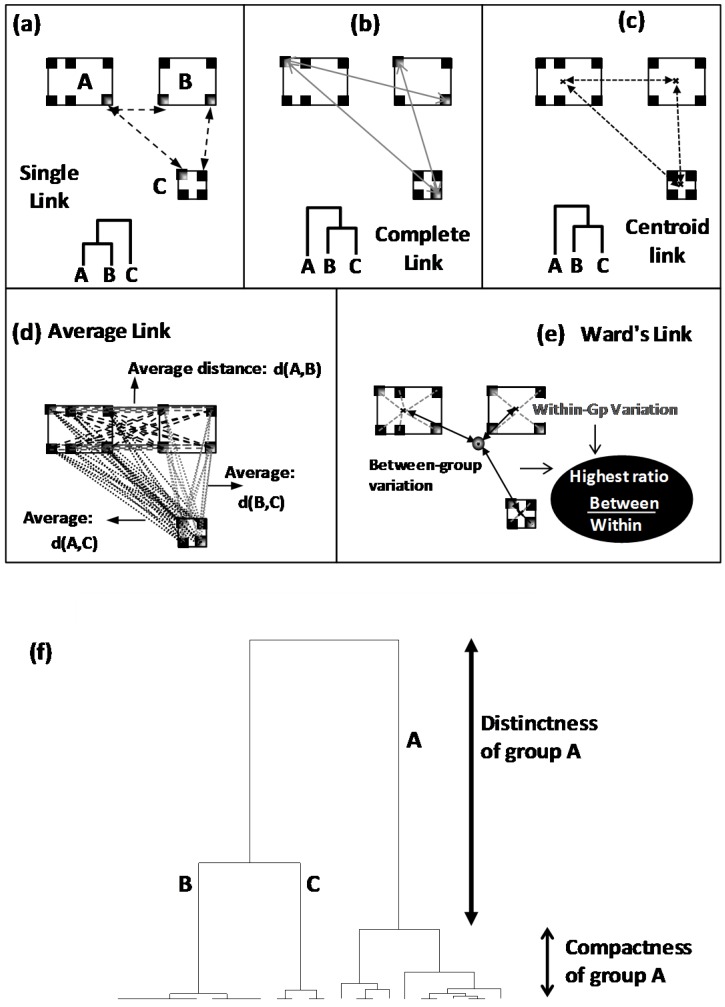
(**a**–**e**) Different agglomeration rules used in hierarchical cluster analysis (HCA); (**f**) Dendrogram or tree-like diagram provided by HCA showing different class structures characterized by different distinction and homogeneity levels (distinctness and compactness, respectively).

Hierarchical cluster analysis (HCA) is a classification method used to organize a whole heterogeneous population into well-distinct and homogeneous groups (called clusters) [20]. By this way, authentic groups are firstly defined as clusters which are more or less separated by well calculated distances: distances are calculated between individuals the closest of which are grouped into same clusters, whereas distant ones are separated into different clusters. In a second technical step, resulting neighbour clusters are merged by applying a given aggregation rule among several possible one: Combination between distance kind and aggregation rule results in several possible tree-like typologies called dendrograms (Figure 4). 

In a third step, the different clusters given by dendrogram are characterized by multivariate patterns due to differential variations and/or non-overlapping variation ranges of different experimental parameters. Finally, dendrogram helps to identify how many distinct groups are constitutive of a whole population on one hand, and how much these groups are close/distant the ones to the others. This usefully helps to understand the organization of whole studied population.

#### 3.1.2. Methodological Steps

The first methodological step in HCA consists in calculating distances or similarity levels between individuals. Distances are calculated when data are quantitative, whereas similarity indices are calculated when the system is described by qualitative variables [20,21,22,23,24]. There are several types of distances that can be used including Manhattan, Euclidean, Chi-2, etc. [20,25].

Similarity indices are also numerous including Sorensen-Dice, Jaccard, Simpson, Tanimoto coefficients [11,20].

After distance calculation, all the individuals will be iteratively grouped or separated into different clusters using an aggregation or linkage rule. These rules include single-, complete-, centroid-, average- and Ward-link clustering [20,21,22,23,24] (Figure 4):

- In single link clustering, neighbour clusters are those having the closest individuals (Figure 4a).
- Complete link clustering proceeds by joining clusters having the less distant extreme individuals (Figure 4b).
- In centroid link clustering, neighbour clusters are those having the closest gravity centres (Figure 4c).
- In average link clustering, an average distance is calculated from all the distances separating all the pairwise of points. Clusters showing the lowest average distance will be merged (Figure 4d).
- Ward linkage is based on the calculation of ratio between two variances: variance between clusters on variance within group. Clustering leading to the lowest reduction of this ratio will be applied (Figure 4e).

From dendrogram, different clusters are identified and interpreted on the basis of two criteria: distinctness between clusters and compactness within clusters (Figure 4f). Distinctness defines the distance separating a cluster from the rest (Figure 4f). Higher distinctness indicates a more distinct (differentiated) cluster. Compactness defines the highest distance existing within a cluster (Figure 4f). Lower is the compactness more homogeneous is the cluster.

#### 3.1.3. Application of HCA in OO Field

HCA was applied to classify many populations of olives using different metabolic and physical-chemical variables.

HCA was applied on phenol, triacylglycerol and sterol contents to statistically highlight the key role of these metabolites in chemical differentiation of five Tunisian minor olive cultivars [26]. 

Also, HCA based on Manhattan distance and complete linkage was used to classify and calculate separations between five Italian OO cultivars on the basis of their FA contents [27]: Both dendrogram and PCA scores plot showed that *Coratina* and *Oliarola* cultivars were the most distant leading to their strong differentiation. The three other EVOOs (*Simone*, *Olivastro*, *Leccino*) showed intermediate states with some co-occurrence clusters in HCA or overlapping subspaces in PCA.

HCA was also applied on 31P NMR data using Euclidean distance and single linkage to explore similarity and dissimilarity between 59 samples representing three OO groups: 34 Greek extra virgin OOs (EVOO), 13 refined OOs (ROO) and 12 lampante OOs (LOO) [28]. Higher homogeneity (identity) was highlighted for EVOO followed by ROO then LOO. 

**Figure 5:**
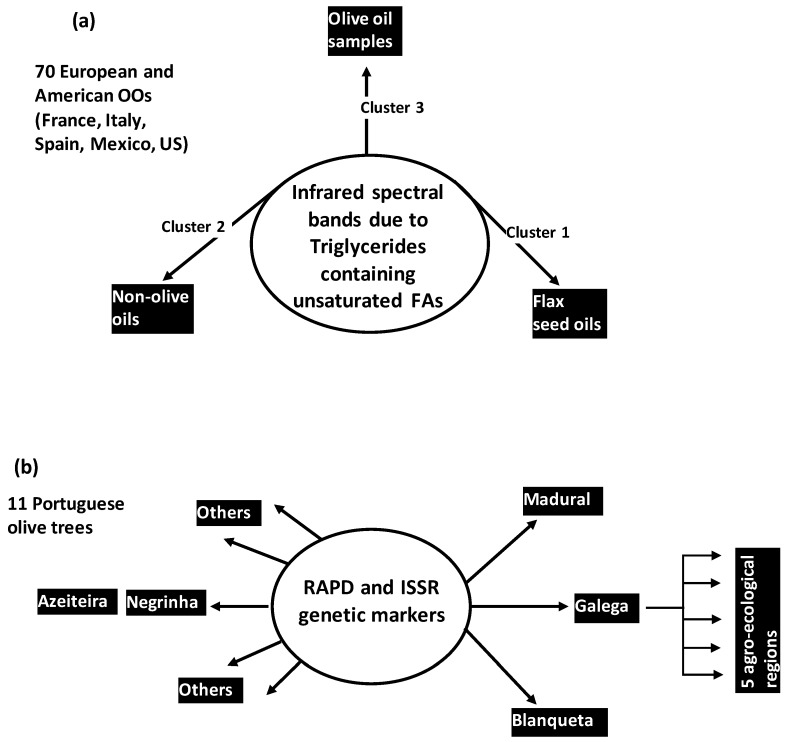
Studies cases on authenticity olive oils (**a**) and olive trees (**b**) highlighted by cluster analysis using spectroscopic (**a**) and genetic (**b**) variables.

In another HCA-based work, OO samples were reliably differentiated from non OO ones on the basis of ATR-FTIR spectra [17]. In this work, 111 pure oil samples were considered including 41 edible vegetable oils and 70 OOs originated from European and American countries (Italy, France, Spain, US, Mexico). The 111 samples were characterized by 2584 wavenumbers covering a spectral region from 650 cm^−1^ to 3588 cm^−1^. Using a variance-weighted distance and Ward’s aggregation method, a dendrogram was constructed to highlight three clusters separating OO samples from two other oil categories (Figure 5a): a first and second clusters represented flax seed oils and most of non-OO samples, respectively. They were frankly separated from a third cluster made by the 70 OO samples in addition to four non-olive samples (one high oleic sunflower, one safflower, two peanut oils). This suggested that some non OO have more similar physical-chemical profiles with OO than other oils. More generally, the HCA results showed that information contained in infrared spectra can be reliably used for OO distinction from other edible oils like canola, corn, soybean, sunflower oils among others. Spectral bands of triglycerides containing unsaturated FAs provided the main discrimination basis. 

Beyond authenticity analysis applied at the scale of separated OO and non-OO samples, the same authors applied HCA to carry out classification of pure OO and binary samples mixing OO with another vegetable oil at variable percentages varying from 10% to 90% [17]. Co-occurring vegetable oils and OOs were concerned with eleven European and American origins. Blends were characterized by infrared (IR) data. Dendrogram obtained with Ward’s method highlighted clear separation between pure OO, non-OO and mixed OO-vegetable oil samples.

Beyond physical-chemical and metabolic markers, HCA was applied on genetic markers (RAPD and ISSR) for inter- and intra-cultivar classification of Portuguese Olive trees [29]. Inter-cultivar dendrogram was obtained using Dice coefficient. It highlighted different similarity-dissimilarity degrees between the eleven studied olive cultivars (Figure 5b): *Galega*, *Madural* and *Blanqueta* cultivars were more dissimilar from all the others, whereas *Negrinha* and *Azeiteira* were the most similar between them. The *Galega* cultivar was then subjected to intra-cultivar HCA using Jaccard index. Resulting dendrogram highlighted five clusters which corresponded to five agro-ecological regions of OO production.

## 4. Chemometric Analysis of Authenticity and Traceability

Beyond authenticity which focuses on identification and origin determination of samples, traceability refers to the quantitative evaluation and qualitative discrimination of components in complex matrices. In OO field, traceability concerned adulteration detection of labelled products and composition evaluations of heterogeneous or multi-varietal blends. A common authenticity-traceability problem concerned the prediction of geographical origins of mono-varietal samples. It is generally treated by linear discriminant analysis among other pattern recognition methods.

Pattern recognition methods include LDA, quadratic discriminant analysis (QDA), stepwise discriminant analysis (SDA), Soft Independent Modelling of Class Analogies (SIMCA) and combining approach based on partial least-square regression and DA (PLS-DA) [30]. For a given profile, these methods calculate several scores corresponding to all the possible cultivars; then the maximal score is retained to attribute the unknown profile to appropriate affiliation group.

### 4.1. Linear Discriminant Analysis 

#### 4.1.1. General Principle

Linear discriminant analysis (LDA) belongs to a set of statistical methods called pattern recognition methods. These methods are applied to predict class membership (e.g., cultivars, geographical origins, etc.) of unknown samples (individuals) from quantitative profiles made by several measured variables (e.g., metabolic concentrations). This goal is reached by constructing a statistical model combining *p* quantitative variables *X_j_* $\left(j_{1}^{p}\right)$ to distinguish between *q* patterns (*q* classes).

#### 4.1.2. Methodological Steps of LDA

Attribution of quantitative individual profiles to appropriate classes requires several methodological steps: patterns’ space is initially constructed, then transformed to reduced discriminant space in which individuals will be finally classified [31].

**Figure 6:**
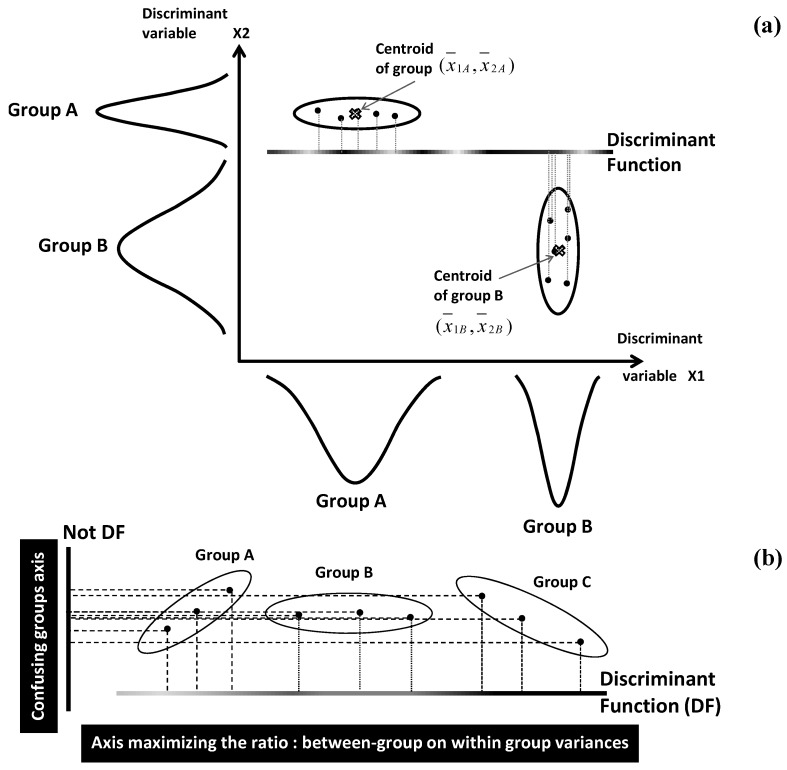
(**a**) Separation of individual points into well distinct groups by a discriminant function combining discriminant variables *X_j_*; (**b**) Comparative illustration of discriminant and not-discriminant axes; linear coefficients in discriminant function are determined by maximizing the ratio of variance between groups on variance within group.

Pattern space construction is carried out by characterizing each group (class) by its centroid position and dispersion range: for a *q*-group system characterized by *p* quantitative variables *X_j_*, centroid of group *k* is spatially located by the average vector: $C_{k}=({\bar{X}}_{1k},\ldots ,{\bar{X}}_{jk},\ldots ,{\bar{X}}_{pk})$. Internal dispersion of a group is given by its variance. For the simplistic example of *q* = 2 groups (A and B) and *p* = 2 variables *X*_1_, *X*_2_, spatial locations of the two centroids are defined by the points of coordinates $\left({\bar{x}}_{1A},{\bar{x}}_{2A}\right)$ and $\left({\bar{x}}_{1B},{\bar{x}}_{2B}\right)$ (Figure 6a). Distances between centroids, and distances of individuals to their centroids are used to define two types of variances called between- and within-classes variances, respectively.

In a second step, the initial pattern space will be transformed by means of linear combinations of the *p* variables *X_j_* $\left(j_{1}^{p}\right)$ [31,32]. Each of the linear combinations provides a linear discriminant function (DF) which represents a separating axis between the *q* groups in the new discriminant space (Figure 6a). 

The coefficients *w_j_* of the different variables *X_j_* in the different linear combinations are optimized by maximizing the ratio of between-group variance on within-group variance. This constrains the *q* groups to be as distinct as possible within a reduced space showing minimum loss of differentiation between the groups (Figure 6b).

In a third step, DFs are used to calculate *q* classification scores (CSs) for each individual. The highest score among the *q* ones will define the appropriate affiliation class of the considered individual. The *q* CSs can also be converted into *q* probabilities among which the maximal value will be considered to attribute the new individual to the most plausible group [33].

#### 4.1.3. Applications of LDA in OO Field

**Figure 7:**
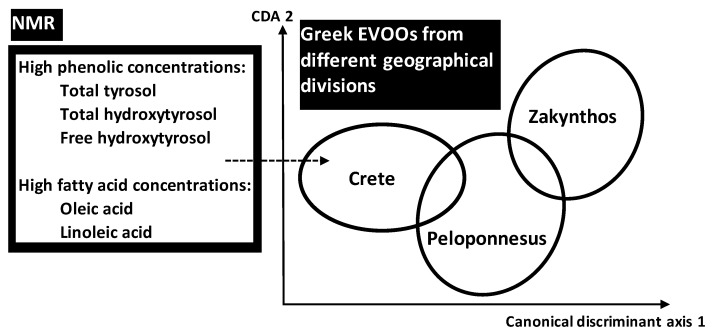
Chemical discrimination of different Greek extra virgin olive oils (EVOOs) originated from different geographical sites of three divisions (Crete, Peloponneus, Zakynthos) using NMR-analysed fatty acids and phenolic compounds.

LDA was widely applied for chemical authentication and traceability analysis of different OO classes including mono-varietal VOOs or EVOOs, registered designations of origins (RDOs), geographical area- and harvesting years, etc. Authentications were based on various discriminant variables including FAs, sterols, triacylglycerols, phenols, volatiles [33,34,35,36,37,38,39]:

Using NMR data of phenolic and FAs compositional parameters, Petrakis et al. (2008) [37] applied discriminant analysis for pattern recognition of different Greek EVOOs taking into account different spatio-temporal scales: (i) three geographical divisions (Crete, Peloponnesus, Zakynthos) including (ii) six sites of origins (Sitia, Heraklion, Chania, Messinia, Lakonia, Zakynthos) from which (iii) 131 samples were obtained at different harvest years. Some metabolites seemed to play important role in discrimination of EVOOs associated with geographical location: the three geographical sites of Crete division were highly discriminated, with frank separation from Zakynthos division (one site) and intermediate state for Peloponnesus division (two sites) (Figure 7). 

The high discrimination of Cretean sites was due their higher total concentrations of total and free hydroxytyrosol and total tyrosol. In addition to phenolic compounds, linoleic (LA) and oleic (OA) acids provided significant markers of geographic origin. Traceability performance was linked to two genes OeFDA2 and OeFDA6 encoding the key enzymes for LA and OA pathway. These genes seem to have survived the extensive gene flow. In Mediterranean olive trees, the gene flow seemed to have obscured the phylogenetic signal but not the geographic one.

LDA was also applied on ^1^H NMR spectra of phenolic extracts to reliably separate between Italian OOs according to both cultivar types and geographical origins [35]. In another work, LDA concerned mass-spectrometry and UV data to differentiate between three geographical nominations of Italian (Ligurian) EVOOs [40]. 

**Figure 8:**
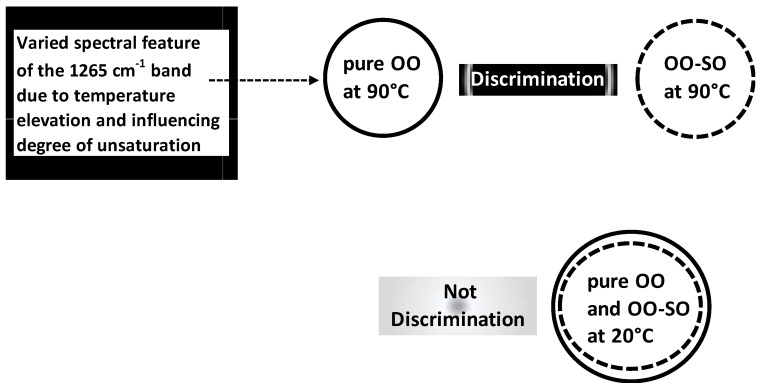
Physical-chemical discrimination of pure OO and adulterated OO oil under temperature of 90 °C due to variation of spectral feature of 1265 cm^−1^ manifesting under temperature elevation.

Beyond authenticity of separated OO samples, LDA was applied for authenticity analysis of OO blends. A work was carried out on normalized Raman spectral in order to discriminate between pure OO, pure soybean oil (SO) and OO-SO blends submitted at different temperatures (20–90 °C) [41]. Pure OO and OO-SO blends (i.e., adulterated OO) under 90 °C were frankly well-discriminated by LDA compared to those under 20 °C (Figure 8); this highlighted interactive discriminant effect linked to temperature elevation. This temperature-dependent effect was associated with varied spectral features of the 1265 cm^−1^ band which was related to the degree of unsaturation.

**Figure 9:**
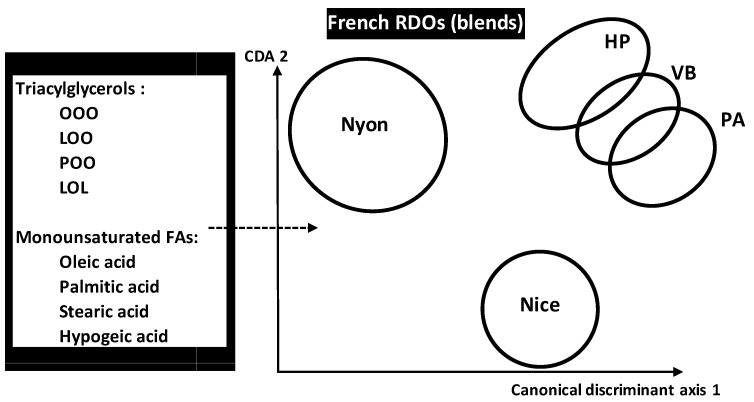
Chemical discrimination of five French VOO registered designation of origins (RDOs) made by mixing different OO cultivars and characterized by 14 fatty acids (FAs) and 19 triacylglycerols (TGC) among which four FAs and four TGCs were the most discriminant. Legend: OOO, triolein; LOO, linoleoyl-dioleoyl-glycerol; POO, palmitoyl-dioleoyl-glycerol; LOL, dilinoleoyl-oleol-glycerol.

OO blends authenticity analysis was also carried out by LDA to predict five French VOO registered designation of origins (RDO) made by combining different cultivars as primary or secondary components. RDOs consisted of *Nyons* (*Y*, *n* = 126 samples), Vallée des Beaux (*VB*, *n* = 98), Aix-en-Provence (*PA*, *n* = 99), Haute Provence (*HP*, *n* = 85) and Nice (*C*, *n* = 131) (Figure 9) [34]. The five RDOs were chemically characterized by FAs and triacylglycerols (TGC) contents (14 FAS and 19 TGCs). Application of LDA on the whole chemical dataset of 539 samples belonging to five RDOs showed that *PA* and *VB* were the closest origins because of minimal Mahalanobis distance separating them. However, *Y* was the most distant RDO flowed by *C* leading to higher authenticity and identicalness. Although all the variables were useful for pattern recognition, some ones revealed to be more particularly influent in RDOs’ discrimination because of higher standardized discriminant coefficients which concerned:

- Triolein (OOO), linoleoyl-dioleoyl-glycerol (LOO), palmitoyl-dioleoyl-glycerol (POO), dilinoleoyl-oleol-glycerol (LOL) for TGCs
- Monounsaturated FAs sum, oleic, palmitic, stearic and hypogeic acids for FAs.

At higher complexity level, LDA was applied on FA profiles to predict multivarietal quantitative compositions of OO blends varying by different weights (proportions) of co-occurring French and Tunisian cultivars [14,16]: such a problematic goes beyond qualitative discrimination of separated cultivars and RDO (blend) types because it makes to evaluate percentages of several co-occurring OOs in same blends from chemical profiles of blends. Application of LDA for prediction of multivarietal OO blends concerned French and Tunisian samples and it will be presented in Section 4.7.3 [14,16].

### 4.2. Partial Least Square Regression

#### 4.2.1. General Principle

PLS can be applied for differential sorting of many input variables according to their pertinence levels for the prediction of the output variable *Y*. This helps for extraction of a small number of reliable predictors of *Y* among a huge mass of many candidate input variables *X*. This is particularly interesting for datasets containing few individuals and many variables [42,43].

Moreover, PLS is a favourite method of treatment with possibly correlated predictors which are combined and condensed with all the initial variables into new composite variables called latent variables (LVs) (or components or factors). 

Like the principal components (in PCA or CA), LVs are newly constructed variables which weight and condense the initial variables to handle with their highly diversified and/or correlative aspects. However, in PLS regression (PLSR) differs from PC regression (PCR) by the fact it incorporates information on both *X* and *Y* in the definition of LVs:

- In PLSR, LVs are calculated such that the covariance between *Y* and *X* is maximal. By this way, LVs are built on the basis of both the input (*X*) and output (*Y*) variables.
- In PCR, one focuses on maximization of the variance of *X* in different spaces. Only the input variables *X* are used for LVs construction.

#### 4.2.2. Methodology of PLS Regression

Initially, PLS proceeds to decomposition of predictors’ matrix *X* into orthogonal scores *T* and loading *P*:

*X* = *T*·*P*

This makes to regress the output variable *Y* on the first columns of the scores instead of *X*. Therefore, prediction of the output variable *Y* by PLSR is carried out iteratively by adding a single LV at each step. By this way, several enclosing PLSR models are obtained to predict the output variable *Y* through linear functions of the first *k* LVs. The best model can be concluded from a minimal prediction error obtained after cross-validation.

The final PLS model has the double advantage to:

- Predict the output variable *Y* from a small number of LVs.
- Help for identification of the most contributive output variables (e.g., spectral wavenumbers).

Thus, PLSR offers an efficient chemometrical tool for variable selection leading to extract the most pertinent predictors of complex and highly variable system such as adulterated OO blends. Before quantitative evaluation of adulteration level (proportion), LDA can be applied to determine the nature of adulterant.

#### 4.2.3. Application of PLS Regression in OO Field

Olive oils can be adulterated by adding some cheap vegetable oils at different proportions in the commercial blends. Such adulterations are qualitatively and quantitatively analysed by means of spectroscopic features including near-infrared (NIR), mid-infrared (MIR) and Raman data. These techniques provide highly condensed information on the mixture matrices through large numbers of recorded wavenumbers (hundreds or thousands of variables). 

Many chemometrical works were carried out by treating IR data by PLSR in order to predict the adulteration level of commercial EVOOs by other vegetable oils added at variable proportions. The list of vegetable oils concerned with adulteration includes: canola, hazelnut, pomace, sunflower, corn, soybean, walnut, etc. [6,44,45,46,47,48,49].

### 4.3. PLS-DA Approach 

#### 4.3.1. General Principle 

PLS-DA refers to a method combining partial least square (regression) and linear discriminant analysis. Like LDA, it aims to optimize separation between different groups characterized by several quantitative variables [50]. It differs from LDA in that the *q* groups are initially represented in *q* separated indicator columns (0, 1), whereas in LDA all the groups are represented by one column containing *q* labels (Figure 5). Therefore, optimization of separation between groups is carried out by linking the (1, 0)-binary matrix *Y* to quantitative matrix *X* containing *p* discriminant variables.

#### 4.3.2. Methodological Steps of PLS-DA 

**Figure 10:**
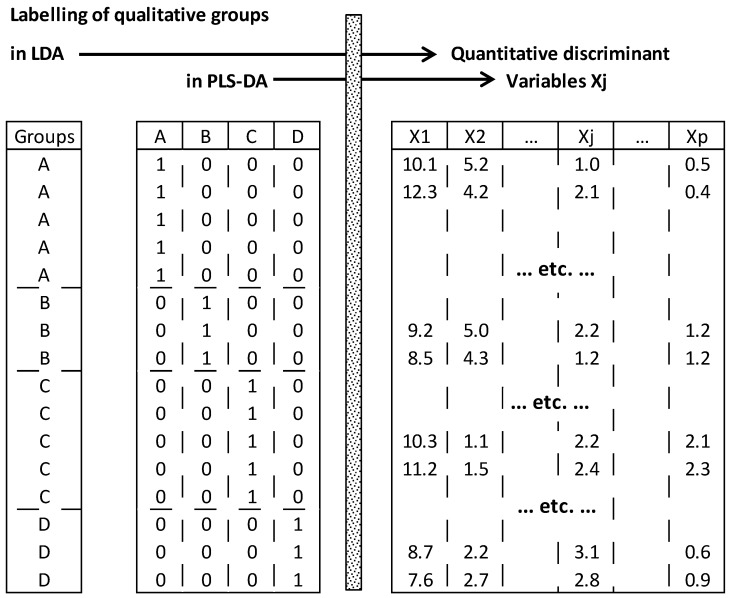
Difference between linear discriminant analysis (LDA) and partial least square regression-discriminant analysis (PLS-DA) based on different initial labelling of groups.

Initially, *q* class membership columns are prepared by taping 1 or 0 if individuals belong or not to corresponding classes, respectively. Then, optimization of separation between the *q* groups is performed by maximizing the covariance between the *p* discriminant variables *X_j_* and the *q* candidate groups (Figure 10). 

Space reduction is carried out by calculating integrative variables, called latent variables (*LVs*), from linear combinations of *X* (*LV_X_*) and *Y* (*LV_Y_*), respectively. Coefficients of linear combinations are calculated under the maximization constraint of covariance of *LV_X_* and *LV_Y_*. This leads to define a linear classifier which has proved to be statistically equivalent to that of LDA, but with noise reduction and variable selection advantage [51]. 

Finally, PLS-DA model predicts continuous values of *Y* variables which are comprised between 0 and 1. Values closer to 0 indicate that the individual does not belong to corresponding classes; however, closer the value to 1 higher the chance of individual to belong to the concerned class. Therefore, individuals are assigned to the classes which have the maximum score in the *Y*-vector. Alternatively, threshold values (between 0 and 1) can be defined for the different classes using Bayes theorem [51].

#### 4.3.3. Application of PLS-DA in OO Field 

PLS-DA models were applied on spectroscopic data (MIR, NIR, FTIR, ^1^H NMR) to predict authenticity and traceability of different EVOOs, protected designation of origins (PDO), registered designation of origin (RDO) and geographically-linked samples [52,53,54,55,56]. 

**Figure 11:**
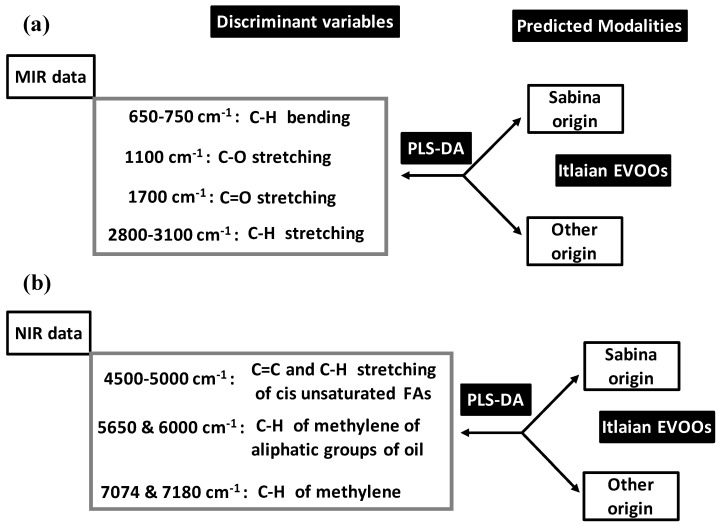
Prediction of geographical origin of Italian EVOOs by PLS-DA using mid- (MIR) (**a**) and near- (NIR) (**b**) infrared data.

Bevilacqua et al. (2012) [52] applied PLS-DA on MIR and NIR data for pattern recognition of geographical origins of Italian EVOOs shared between Sabina and not Sabina (confounded other sites) (Figure 11). 

Before PLS-DA, MIR and NIR data were subjected to different pre-treatment for baseline corrections consisting of: (a) linear; (b) quadratic (Q); (c) multiplicative scatter (MS); (d) detrending (D); (e) first derivative (1d); (f) second derivative (2d); (g) MS with 1d; (h) MS + 2d; (i) MS + Q; (j) MS + Dt. NIR and MIR datasets issued from pre-treatments were modelled using calibration (Cbr) and cross-validation (CrV) techniques.

Using MIR data, the best predictive PLS-DA model was built by MS pre-treatment (followed by QB). This model predicted Sabina at 100% and 92.3% in Cbr and CrV vs. 95% for other origin in both Cbr and CrV. Moreover, outside samples were given to PLS-DA model (for final validation) which predicted Sabina and other origin at 85.7% and 86.7%, respectively. Analysis of discrimination ability of MIR variables showed that C=O double bond stretching (around 1700 cm^−1^) and C–H bending (within 650–750 cm^−1^ range) contributed the most to the PLS-DA model (Figure 11a). They were followed by significant (but lower) contributions of C–H stretching (2800–3100 cm^−1^) and C-O (single bond stretching) (1100 cm^−1^).

Using NIR data, four PLS-DA models showed high predictive aspect both in Cbr and CrV. They concerned MS, Dt, 1d and MS + D which predicted Sabina and other origin at 100% and 95.5%, respectively. Final validation based on outside sample was concluded by predictive ability of: (i) 100% for both Sabina and other origin with 1d-based model; and (ii) 100% Sabina vs. 93.3% other origin with the three other models. Discriminant variables included all the spectral regions (Figure 11b):

- 4500–5000 cm^−1^ due to combination bands C=C and C–H stretching variation of cis unsaturated FAs.
- 5650 and 6000 cm^−1^ due to combination bands and first overcome of C–H of methylene of aliphatic groups of oil.
- 7074 and 7180 cm^−1^ linked to C–H band of methylene.

Apart from DA, PLS was combined with linear regression (PLSR) to predict proportions (0% to 5%) of EVOO and Palm oil (PO) in three vegetable oil blends based on corn oil (CO), soybean oil (SO) and sunflower oil (SFO), respectively [4]. EVOO and PO represent desired and undesired (adulteration) oils in vegetable oil blends. Blends were initially characterized by UV spectra containing many wavelength-absorbance variables. The high number of these variables was reduced by applying PCA which provided principal components used as latent variables in PLSR. PLSR models predicted EVOO proportions in EVOO-vegetable oil blends with determination coefficient *R*^2^ = 0.86, 1.00 and 0.98 in EVOO-CO, EVOO-SO and EVOO-SFO blends, respectively. For adulterant PO in EVOO-vegetable oils, PLSR predicted its proportions with *R*^2^ = 0.85 (in EVOO-CO), 1.00 (in EVOO-SO) and 0.99 (in EVOO-SFO).

### 4.4. Soft Independent Modeling of Class Analogies

#### 4.4.1. General Principle

Soft Independent Modelling of Class Analogies (SIMCA) is a pattern recognition method commonly used in chemometrics. The term “Soft” means that no hypothesis on the distribution of variables is made. This concept makes that overlapping of classes is not problematic, and individuals can be flexibly affiliated to more than one class. Flexible solution is also provided by the concept of independency where the classes are separately modelled. Specific models of different classes are made by applying PCA on each class. At the end, each class will be modelled by a specific PC-based model which will delimit it in a region of space. A new individual will be affiliated into class *k* if its distance to corresponding region *k* is lower than those to other regions (classes).

#### 4.4.2. Methodological Steps

**Figure 12:**
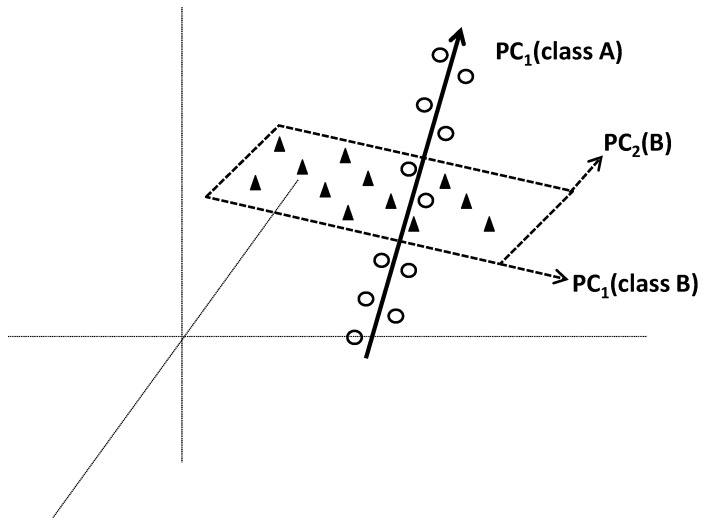
Illustration of the principle of Soft Independent Modelling of Class Analogies (SIMCA) based on independent application of principal component analysis on each class. In the example, class A is well predicted by the first PC _PC_1_(A)_ whereas class B needs the first two PCs _PC_1_(A) and PC_2_(B)_ to be well predicted.

From each separately applied PCA, a class model is built from PCs that best fit the variation within corresponding class. The number of significant PCs in each class-model can be determined by cross-validation. Figure 12 illustrates two class models based on one and two PCs, respectively.

The collection of *q* models will define *q* subspaces characterizing the *q* classes. Projection of new individual *i* is followed by the calculation of *q* geometric distances to the *q* subspaces. The distance *d_ik_* between individual *i* and class *k* is calculated by a combination of the distance within the model space (*T*^2^ statistics or leverage) and orthogonal distance to the model space (*Q*^2^ statistics or squared residuals) [52]. Individual *i* is affiliated to class *k* if *d_ik_* is lower than a threshold value.

#### 4.4.3. Application of SIMCA in OO Field 

SIMCA was applied for pattern recognition of different VOO and EVOO varieties using chromatographic (FAs, triglycerides, sterols) and spectrometric data (NIR, UV, MS, NMR) [15,40,52,54,57,58]. Analogous work using PLS-DA was illustrated in Figure 11.

SIMCA-based models were developed to predict origins of Italian EVOOs issued from Sabina and other sites. This helped for traceability analysis of a protected designated of origin vs. oils from other origins [52]. Several predictive models of Sabina were developed on the basis of NIR and MIR data after different types of baseline corrections. The number of latent variables of each model was determined from the highest geometrical averages of sensitivity and specificity obtained in ten iterative cross-validation. Models built from NIR showed higher sensitivity and specificity than MIR-based ones. According to pre-treatment ways, the best performances were obtained with detrending (D) and multiplicative scatter correction (MS) with first derivative (1d) which both resulted in 100% sensitivity and 95.45% specificity in calibration-based models, and 76.92% sensitivity and 95.45% specificity in cross-validation. Final validation issued from predicted outside patterns gave100% sensitivity and 93.33% specificity under D vs. 71.43% sensitivity and 86.67% specificity under MS with 1d. 

Finally, data analysis by SIMCA vs. PLS-DA showed different optimal pre-treatment way in the two approaches [52]. This was due to the fact that the two methods are differently influenced by the shapes of class distributions. This could be directly linked to the different principles of the two techniques: SIMCA focuses on the similarity of individuals within a same class; the *q* classes are considered separately the ones from the others leading to *q* specific categorical spaces. Finally, with *q* classes, one obtains *q* class-models based on opportune numbers of latent variables selected among all the principal components of a preliminary applied ordination analysis. However, DA (PLS-DA) operates by evaluating the differences between classes; individuals are attributed to appropriate classes by maximizing the ratio of variance between- on variance within-classes. DA operates by decomposing the hyperspace of the variables in *q* subspaces associated to the *q* predicted classes.

In another work, SIMCA was applied to discriminate West Ligurian EVOOs from volatile terpenoid hydrocarbons (VTH) which were analysed by GC-MS in 105 OO samples originated from: Italy (West Ligura, Puglia), Greece, Spain and Tunisia [59]. Eight VTH were separated including α-pinene, limonene, trans-β-ocimene, 4,8-Dimethyl-1,3,7-nonatriene, α-Copane, Eremophyllene, α-Muurolene and α-Farnesene. SIMCA models showed high predictive ability of Ligurian EVOOs vs. each of the six other origins, sensitivity and specificity varying in the ranges 81%–90.9% and 92%–100%, respectively. Finally, among the eight VTHs, α-copaene, α-muurolene and α-farnesene showed high discriminant values in the separation of Ligurian samples from the other geographical origins. 

### 4.5. Support Vector Machines 

#### 4.5.1. General Principle

**Figure 13:**
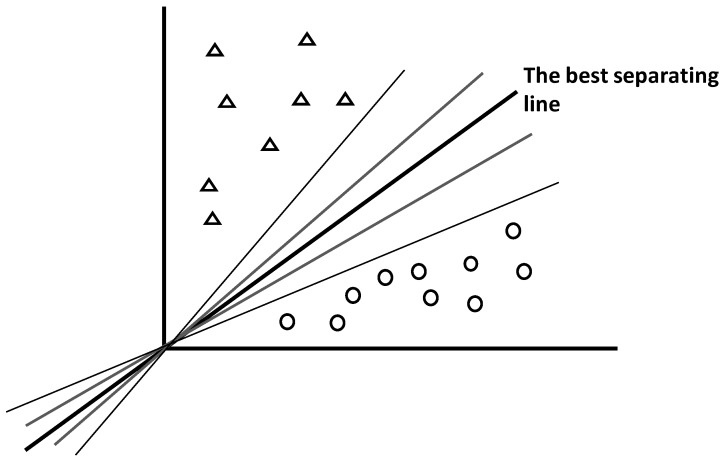
Simplistic illustration of the concept of optimal hyperplane separating two classes (two subspaces) among infinity of possible (not optimal) hyperplanes.

Support vector machines (SVMs) are supervised methods used to attribute unknown features to one among two possible candidate classes (e.g., class A vs. class B for binary system, or A vs. not A for multiclass system) [60,61]. For that aim, SVMs search optimal hyperplane separating well the two subspaces representing the two feature subsets. To be optimal, the hyperplane needs to have appropriate spatial location and angulation (Figure 13).

As shown by the intuitive Figure 13, there are infinity of possible lines separating two subspaces, but only one provides the best separation.

#### 4.5.2. Methodological Steps

**Figure 14:**
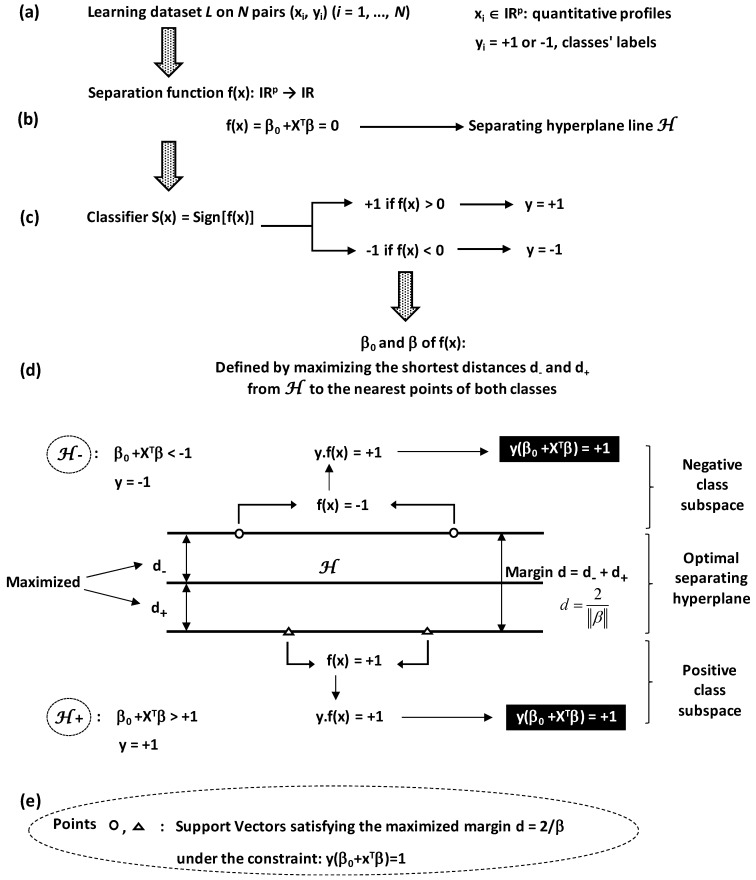
Different components (**a**–**e**) for Support Vector Machines. (**a**) Information training source; (**b**) separating hyperplane function to be defined; (**c**) binary classification rule; (**d**) optimisation condition for determining hyperplane function; (**e**) margin separation limits issued from optimization process.

Construction of optimal separating hyperplane between two classes requires a learning dataset *L* of *N* pairs (*x_i_*, *y_i_*) (*i* = 1, …, *N*) where (Figure 14a):

*x_i_* are quantitative vectors with *p*-dimension (*p* variable features; *x_i_* ∈ $IR^{p}$).

*y_i_* are binary values equal to +1 or −1 depending on the class to which *x_i_* belongs.

Hyperplane is algebraically defined by a separation function *f*(*x*) taking positive or negative real values by the line equation (Figure 14b):

$$f(x)=\beta_{0}+X^{T}\beta =0$$

where *β* is a normal vector to the hyperplane and *β*_0_ is offset. 

The line will serve as reference to attribute any point x to one among the two classes according to the classifier rule *S*(*x*) (Figure 14c):

$$\begin{array}{ccc} S(x) & =\mathrm{sign}[f(x)] & \\ & =+1\mathrm{if}f(x)>0 & \left(\to y=+1\right)\\ & =-1\mathrm{if}f(x)<0 & \left(\to y=-1\right) \end{array}$$

Separation between the two classes is reinforced by establishing two limits at each side of the hyperplane line *f*(*x*) = 0 (Figure 14d). The two limits are defined by the shortest distances *d*_−_ and *d*_+_ from the hyperplane to the nearest points of both classes. They are characterized by the hyperplane lines equations:

$$f(x)=\beta_{0}+X^{T}\beta =-1\mathrm{and}f(x)=\beta_{0}+X^{T}\beta =+1$$

Points located on these limits (the nearest points) are called support vectors (Figure 14e).

Finally, the determination of parameter values *β*_0_ and *β* will be carried out by maximizing the shortest distances *d*_−_ and *d*_+_. These distances satisfy a maximized margin *d* (the hyperplane wide) which is equal to 2/‖β‖ (*d* = *d*_−_ + *d*_+_ = 1/‖β‖ + 1/‖β‖ = 2/‖β‖). Moreover, margin maximization obeys to the constraint $y\left(\beta_{0}+X^{T}\beta \right)=1$ because y = −1 and +1 for $\left(\beta_{0}+X^{T}\beta \right)\leq -1$ and $\left(\beta_{0}+X^{T}\beta \right)\geq +1$, respectively.

By considering all the pairs of points (*x_i_*, *y_i_*) of the binary system, SVMs algorithm corresponds to a convex optimization problem helping to find *β*_0_ and *β* that minimize $\frac{1}{2}{\left\|\beta \right\|}^{2}$ under the constraint: $y_{i}\left(\beta_{0}+{x_{i}}^{T}\beta \right)\geq +1$ ∀ *i* = 1 to *N*.

**Figure 15:**
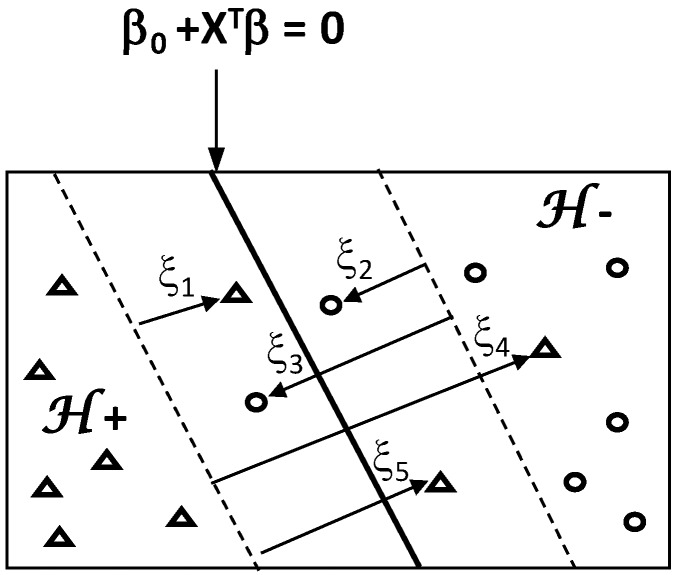
Improvement of separation between classes by SVMs using slack variable *ξ*. The five *ξ_i_* (*ξ*_1_, …, *ξ*_5_) are associated with the points that violate the constraints of hyperplane H+ and H−.

Beyond the basic concept of linearly separable classes, feature recognition can be improved by introducing the concept of a soft-margin based on a more flexible formulation of the problem (Figure 15). This makes to overcome misclassification problem due to overlapping between classes, i.e., infiltration of some points of one class into the space of the other class. 

Under technical aspect, soft-margin between classes is constructed by introducing a nonnegative slack variable *ξ* which takes *N* values for the pairs (*x_i_*, *y_i_*): *ξ* = (*ξ*_1_, …, *ξ*_N_) ≥ 0. The slack variable *ξ* is introduced to allow the violation of the margin constraints of hyperplane. 

Therefore, determinations of *β*_0_, *β* and *ξ* are carried out by solving soft-margin optimization problem:

$$Min\left[\frac{1}{2}{\left\|\beta \right\|}^{2}+C\sum_{i=1}^{N}\xi_{i}\right]$$

Under the constraints:

*ξ*_i_ ≥ 0

and

$$y_{i}\left(\beta_{0}+{x_{i}}^{T}\beta \right)\geq 1-\xi_{i}\;\mathrm{for}\;i=1\mathrm{to}N$$

where *C* is a control parameter of the slack variable sizes (called regularisation meta-parameter). It controls the error of misclassification by compromising between two conflicting objectives: minimizing the training error vs. maximizing the margin. Higher and lower *C* values result in emphasizing the error minimization and the margin maximization, respectively.

Optimization of margin distribution provides significant improvement of class separation in the case of non-separable data in the feature space.

**Figure 16:**
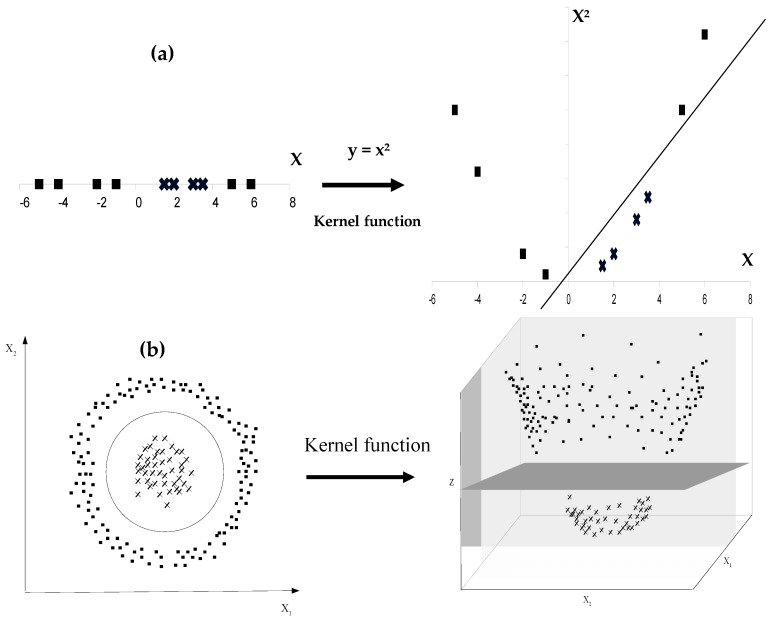
Two illustrative examples (**a**,**b**) showing how two nonlinearly separable classes can be separated in a higher dimension space reached by a data transformation based on a kernel function. The illustrative cases (**a**) and (**b**) correspond to two states requiring two different data transformations (using two different kernel functions) for final linear separation of the two classes.

When two classes are nonlinearly separable, their discrimination by SVM can be performed by using a mapping function ϕ. This function serves as basis of a kernel which transforms the original feature space into a higher dimensional space in which a separating hyperplane can be found to linearly separate the two initially overlapping classes (Figure 16).

In SVM algorithm, this transformation involves scalar products between input vectors $〈\vec{x_{i}},\vec{x_{j}}〉$.

Given *φ* the mapping function: *φ*: $\vec{x}$→ *φ*($\vec{x}$)

So the scalar product is: $k\left(\vec{x_{i}},\vec{x_{j}}\right)=〈\varphi \left(\vec{x_{i}}\right),\varphi \left(\vec{x_{j}}\right)〉$

Where: $k\left(\vec{x_{i}},\vec{x_{j}}\right)$ is called kernel function.

List of kernel functions is wide including polynomial kernel, radial basis function kernel, sigmoid kernel, Pearson VII universal kernel (PUK), etc. In practice, choosing the appropriate kernel is not an easy task, and tuning the parameters of chosen kernel is essential to get good performance from SVM algorithm.

#### 4.5.3. Application of SVMs in OO Field

SVMs were applied to Italian OOs characterized by near- and mid-infrared spectra in order to discriminate between Ligurian and not Ligurian origins [62].

**Figure 17:**
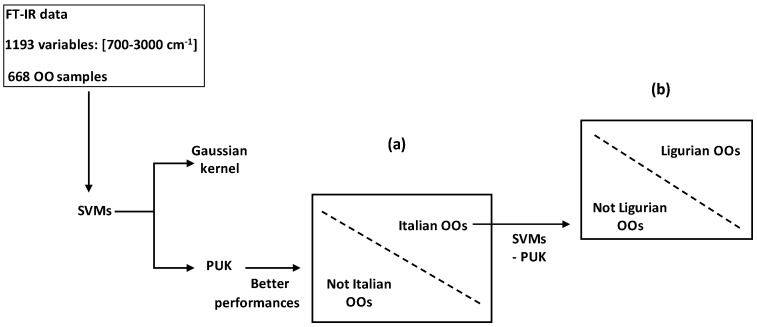
Application of SVMs combined with kernel function to discriminate between (**a**) Italian and not Italian olive oils and between (**b**) Ligurian and other Italian regions using FT-IR data [63]. PUK: Pearson Universal Kernel.

In another work, FT-IR data (input spectroscopic features *x_i_*) were treated by SVMs combined with kernel function to successfully discriminate between (i) Italian and not Italian OOs; and between (ii) Ligurian and other Italian regions (Figure 17) [63]. Comparison between predicted results issued from SVMs combined with Pearson VII Universal kernel (PUK) and Gaussian kernel (GK) showed higher performances of the SVM-PUK method. This made conclusion about the higher mapping power of PUK for discrimination of binary systems by SVMs.

### 4.6. K-Nearest Neighbours 

#### 4.6.1. General Principle 

K-nearest-neighbour method (K-NN) is a pattern recognition method used to attribute new features to appropriate classes among several candidate classes. Due to its nonparametric aspect, it can be fundamentally applied for new patterns classifications under little or no prior knowledge on data distributions [64]. 

Application of K-NN needs a learning dataset containing *N* points representing *N* features which are spatially separated the ones from the others by local distances that can be Euclidean or of other types. The type of used distance depends on the type of data.

**Figure 18:**
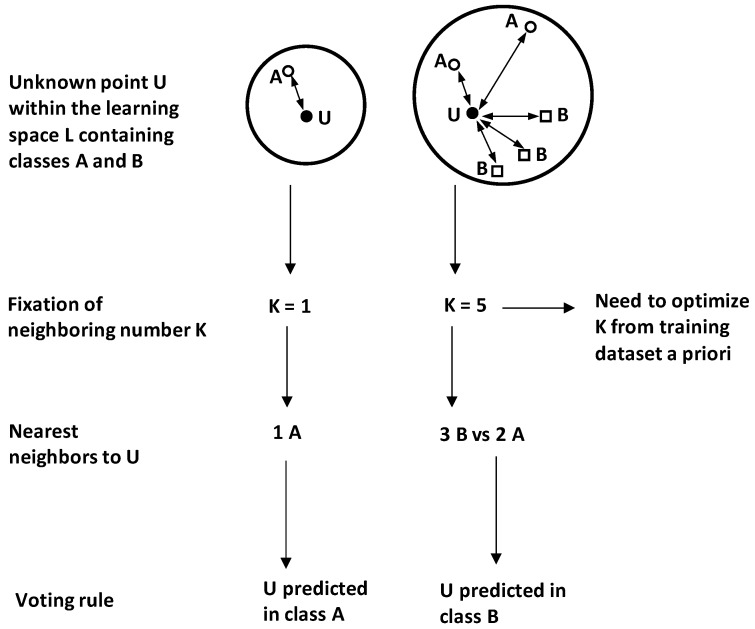
Two examples illustrating the K-Nearest Neighbours principle and the influence of the neighbouring number *K* on classification results. Parameter *K* needs to be optimized.

Attribution of a new point *U* to appropriate class by K-NN is based on the evaluation of its location by reference to its *K* nearest neighbours (Figure 18). This results in *K* calculated distances between the unlabelled (new) point *U* and the *K* nearest neighbours. Therefore, K-NN decision rule consists in attributing U to the class to which most of the *K* neighbours belong.

However, by this majority rule, the *K* nearest neighbours will have equal influence despite their different distances from *U*. For that, standard K-NN can be improved by weighted K-NN by individually considering the *K* distances to attribute different weight values to the *K* neighbours. By this way, higher voting weights are attributed to the closest (less distant) neighbours.

#### 4.6.2. Methodological Steps

Formally, K-NN initially uses a training or learning set *L* defined by *N* multivariate features *x_i_* belonging to *C* classes *y_i_* (*y_i_* = 1, …, *C*):

*L* = {(*x_i_*, *y_i_*), *i* = 1,…, *N*}

where *x_i_* denotes a vector containing *p* predictor variables:

*x_i_^T^* = (*x_i_*_1_, *x_i_*_2_, …, *x_ip_*)

Pattern classification of unlabelled point *U* requires the application of distance function *d* making to evaluate neighbouring or similarity between *U* and *K* points representing its nearest neighbours (*NNs*) among the *N* points of learning dataset. For instance, Minkowski distance provides a general formula giving different desired distances depending on the parameter *q* [65]:

$$d\left(x_{i},x_{j}\right)={\left[{\sum_{t=1}^{p}\left|x_{it}-x_{jt}\right|}^{q}\right]}^{\frac{1}{q}}$$

where *x_i_*, *x_j_* are two features *i* and *j* characterized by *p* variables *x*_1_, … *x_t_*.

The formula provides several types of distances according to the *q* value, including: Euclidean distance for *q* = 2, absolute distance for *q* = 1, etc. To avoid high variance effects, the different variables *x_t_* are initially standardized to mean 0 and variance 1 by applying:

$$\frac{x_{it}-{\bar{x}}_{t}}{SD_{t}}$$

where 

*x_it_* is the value of variable *x_t_* in feature *i*, 

${\bar{x}}_{t}$ and *SD_t_* are the mean and the standard deviation of variable *x_t_*, respectively, initially calculated on raw data.

After calculation of *N* distance values between *U* and the *N* learning points *x_i_*, the lowest values will be kept and associated to the *K* *NNs*. Therefore, the *C* classes will be concerned by different numbers *K_r_* (among *K*) due to the different belongings of the *K* *NNs*:

$$\sum_{r=1}^{C}K_{r}=K$$

Therefore, the new point *U* will be predicted into the class *m* showing majority of neighbours:

$$K_{m}=\max{\left(K_{r}\right)}_{r=1}^{r=C}$$

Note that the predicted class of a new feature *U* can be significantly influenced by the chosen neighbouring number *K* (Figure 18). 

For instance, in the illustrative case of Figure 16x, choosing *K* = 1 makes the unlabelled point *U* to be classified in class A because its nearest single neighbour belongs to *A*. However, but using *K* = 5, the point *U* will be attributed to class *B* because it becomes majority with three points *B* vs. A with two points. 

Therefore, the neighbouring number *K* needs to be chosen among several candidate values using some global minimization error criterion. Moreover, several K-NN algorithms (including weighted K-NN) were developed from the standard one to (i) avoid equal importance for all neighbours and to overcome imbalanced data problems [66].

#### 4.6.3. Application of K-NNs in OO Field

Bajoub et al., (2017) [67] applied K-NN to predict seven EVOO varieties cultivated in Morocco and characterized by phenolic HPLC profiles: *Arbequina*, *Arbosana*, *Cornicabra*, *Frantoio*, *Picholine de Languedoc* (*PL*), *Picholine Marocaine* (*PM*) and *Picual*.

Phenolic fingerprints of the seven varieties (represented by 140 samples in all) were acquired by two types of spectroscopic data consisting of diode array (DAD) and fluorescence (FLD). Detection conditions were 280 nm for DAD and 280 nm (*λ* excitation) and 339 nm (*λ* emission) for FLD.

Application of K-NN on HPLC-DAD dataset provided high prediction accuracy for the seven EVOO cultivars; accuracies ranged within 93.2%–100% for the training set, (91.26%–100%) for the cross-validation and (94.60%–100%) for the test prediction set. The best prediction rates concerned the variety *Cornicabra* whereas the lowest ones concerned *PL* and *PM*.

Using HPLC-FLD data, K-NN models gave discrimination rates >96.12%, 97.09% and 94.60% for training, cross-validation and prediction sets, respectively. The best discrimination results concerned the varieties *Cornicabra*, *Arbequina* and *Arbosana*; the lowest accuracy values concerned *PM* cultivar.

### 4.7. Artificial Neural Networks 

#### 4.7.1. General Principle 

Artificial neural networks (ANNs) represent a set of unsupervised learning approaches used to build non-linear models predicting *q* classes or responses from iterative combinations of *p* weighted control variables [68]. ANNs are sophisticated methods for both classification and prediction providing a visualization of how different categorical variables (e.g., OO cultivars, blends, geographical origins, etc.) are separated in different hyperspace areas with different shapes serving as reference for pattern recognitions of new samples. On this basis, ANNs represent efficient methods for variation absorption and signal separation leading to reliable traceability predictive models.

#### 4.7.2. Methodological Steps of ANNs

**Figure 19:**
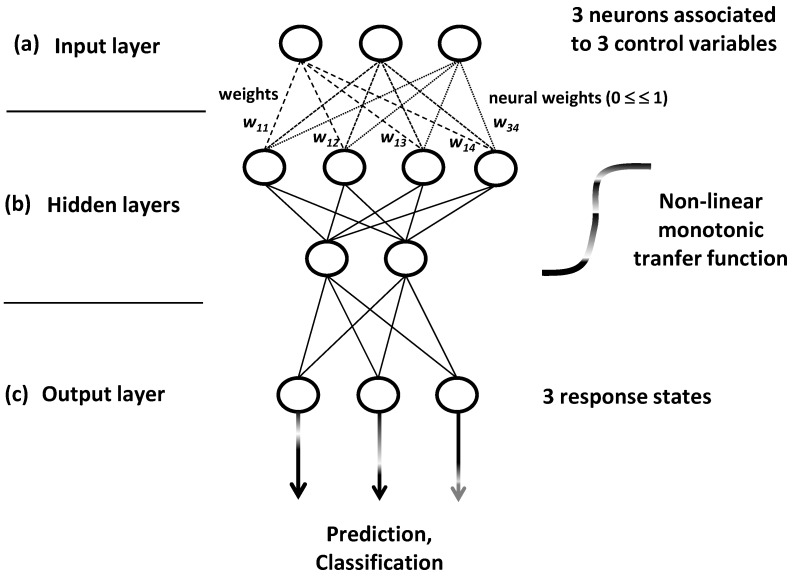
Representation of artificial neural network based on three neural layers (input, hidden, output) (**a**, **b**, **c**) and applied to carry out non-linear predictive models of polymorphic response or state system from different input control variables.

ANN models are built by iterative training algorithms which conceive network systems into three neuron layers: input, hidden and output layers (Figure 19). 

For training process, individuals separately and successively enter in the network as multivariate profiles with *p* learning variables. This input process occurs at the first network layer made by *p* neurons which are associated to the *p* variables (Figure 19a). The system is iteratively trained by several profiles to gradually structure and stabilize it by learning new or confirmed information: the input profiles (with *p* variables) enter in the neural network to bring signals which are detected and categorized by the internal or hidden layers (Figure 19b). Input and cumulative information are transformed by a non-linear monotonic transfer function giving response weight values comprised between 0 and 1.

The set of response weight values iteratively vary until the formation of confirmed specialized fields. Such fields reveal to be sensitive toward different signals or categories to which different individuals can be affiliated. Finally, the stable system structure reveals to be influenced by non-linear links between the *p* input control variables and the *q* revealed output states of systems.

Finally, the *q* categorized and confirmed signals are received by the output layer made by *q* feature-collecting or responses-producing neurons (Figure 19c).

#### 4.7.3. Application of ANNs in OO Field 

ANNs have been applied for classification of 572 Italian OOs from FAs profiles (input variables) [69]. In another work, geographical traceability of different European VOOs (Spain, Italian, Portugal) was modelled in relation to FAs profiles [70]. In the same work, predictions of regions, provinces and PDOs required more input variables including sterols, alcohols and hydrocarbons in addition to FAs. 

**Figure 20:**
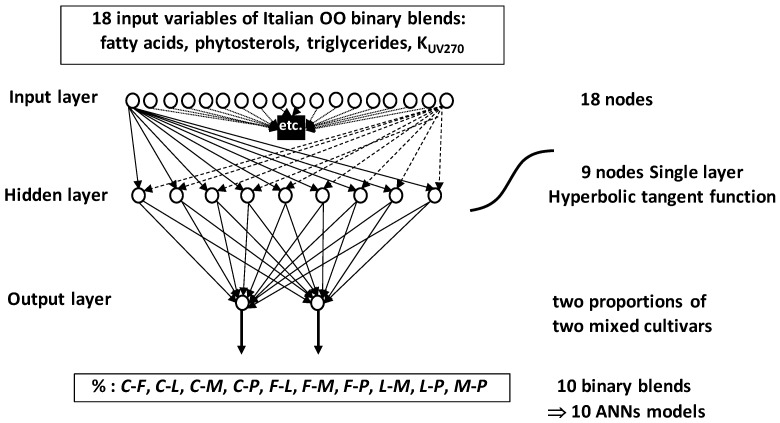
Prediction of proportions of Italian OO cultivars’ pairwise combined in ten different blend types by artificial neural networks (ANNs) using physical and chemical measured variables.

At more complex level, ANNs were used to predict levels (proportions) of OOs in bi-varietal blends [71,72] (Figure 20). 

Coupling two ANNs methods (self-organizing map and multilayer feed-forward), Marini et al. (2007) [72] developed predictive models giving proportions of five Italian mono-cultivars OOs co-occurring in different binary mixtures. The five OO cultivars were *Carboncella* (*C*), *Frantoio* (*F*), *Leccino* (*L*), *Moraiolo* (*M*) and *Pendolino* (*P*). They were chemically characterized by relative levels of different FAs, phytosterol and triglyceride in addition to UV extinction coefficient K_270_. These variables resulted in 18 measured or calculated characteristics which were used as 18 unit signals (18 nodes or neurons) in the input ANNs-layer. Ten binary mixture types were simulated by combining 153 samples representative of the five mono-cultivar OOs leading to several thousands of bivarietal blends of types: *C*-*F*, *C*-*L*, *C*-*M*, *C*-*P*, *F*-*L*, *F*-*M*, *F*-*P*, *L*-*M*, *L*-*P*, *M*-*P*. Optimization analysis initialized with 6 to 15 nodes made determination of the best number of nodes to 9 within a single hidden layer. Preliminary studies showed the not need to add more than a single hidden layer. Finally, the output layer consisted of two nodes providing the proportions of the two mixed cultivars in the considered binary blend. Training was performed by the back-propagation algorithm using a hyperbolic tangent transfer function. The ten ANNs models associated to the ten simulated binary blends predicted the percentages of different OO cultivars’ pairwise with good accuracy indicated by high cross-validation determination coefficients *Q*^2^ (*Q*^2^ = 0.91–0.96).

Cajka et al. (2010) [39] applied ANNs with multilayer perceptron (MLP) and back propagation to predict geographical origins of Ligurian and non-Ligurian VOOs from volatile compounds patterns. In all, 44 volatiles were analysed by GC-MS including alcohols, aldehydes, ketones, esters, carboxylic acids and hydrocarbons. Olive oil dataset consisted of 210 Ligurian and 704 non-Ligurian samples originated from different countries (Italy, Spain, France, Greece, Cyprus, Turkey).

A preliminary PCA on the dataset (914 samples × 44 volatiles) showed wide distribution and overlapping between different OO samples. LDA applied for prediction of Ligurian origin gave prediction ability of 61.7%. This relatively low value was significantly improved by applying ANNs which provided an advanced chemometric tool to treat systems with complex variability and relationships between predictor and predicted variables.

The ANNs model consisted of input, hidden and output layers containing 44, 25 and 1 neuron(s), respectively. The 44 input neurons corresponded to the 44 volatiles, whereas the single output neuron was reserved for geographical origin prediction (Ligurian vs. non-Ligurian). ANNs model showed recognition ability of 91.4% and 84.0% for training and selection subsets, respectively. External validation on test subset showed prediction ability of 81.1%, with a sensitivity and specificity of 84.1% and 80.7%, respectively (vs. 89.9% and 58.2%, respectively in LDA).

### 4.8. Simplex Mixture Networks

#### 4.8.1. General Principle

Simplex mixture networks are particularly advantageous for quantitative control of compositions of mixture systems. Beyond binary systems, simplex spaces are particularly appropriate for variability analysis of high dimension-mixtures (e.g., multivarietal blends). They provide robust way for analysis and control of proportions of several (*q*) co-occurring components (e.g., OO cultivars) in complex blends. In this framework, a new simplex-based approach was developed to predict proportions of mixed groups from quantitative multivariate profiles of their blends [14,16,73]. 

**Figure 21:**
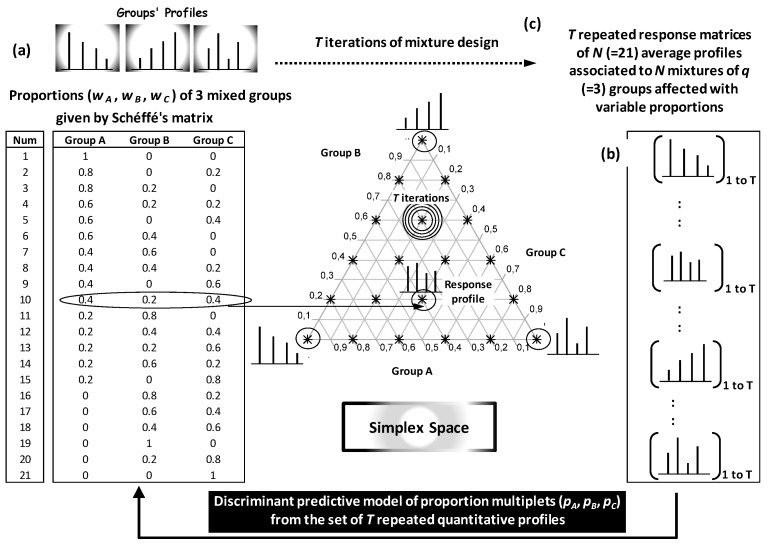
Principle and parameters of simplex approach applied to predict proportions of different mixed groups from quantitative profiles of resulting blends. (**a**) Stratification of OO samples; (**b**) application of Scheffé’s mixture design and calculation of response matrix; (**c**) iterative application for training process before building of proportions’ predictive model.

Theoretically, *N* blends can be represented by *N* combinations of *q* groups *k* (*k* = 1 to *q*) with *q* proportions *w_k_* linked by the unit sum rule Σ*w_k_* = 1. The weights’ multiplet (*w*_1_, …, *w_k_*, …., *w_q_*) represents *q* coordinates attributing corresponding blend to a well-defined spatial location within a simplex space (Figure 21). The simplex approach was developed to predict the weights’ multiplets of different blends from characteristic quantitative profiles. Model construction requires a training step in which each mixture point need to be learned by a large set of representative blend profiles. This implies statistical exploration of variability between and within groups.

#### 4.8.2. Methodological Steps of Simplex-Based Approach

In simplex-based approach, each blend *i* combining *q* groups (*q* OO cultivars) is initially represented by a row *i* with *q* weights’ columns (*w*_1_, …, *w_k_*, …., *w_q_*)*_i_* (*i* = 1 to *N*). The set of *N* possible blends is given by a mixture design called Scheffé’s matrix (Figure 21a) [11,14,74]. This matrix combines *q* groups according to gradually variable proportions using initially a defined increment. 

At the output of mixture design, *N* average profiles are calculated to represent *N* gradually variable blends due to gradual weighting of the *q* groups (Figure 21b). This elementary scheme is iterated *T* times by bootstrapping in order to train the response matrix of *N* average profiles by inter- and intra-group variations (Figure 21c). 

The set of *T* response matrices representing (*T* × *N*) average profiles is finally used to build predictive models of groups’ weights (e.g., OO cultivars’ proportions) from quantitative profiles of blends (e.g., chromatographic profile).

By considering each blend as a multiplet of weights, predictive model of weights from quantitative profiles can be carried out by means of discriminant analysis.

#### 4.8.3. Application of Simplex-Based approach in OO Field

**Figure 22:**
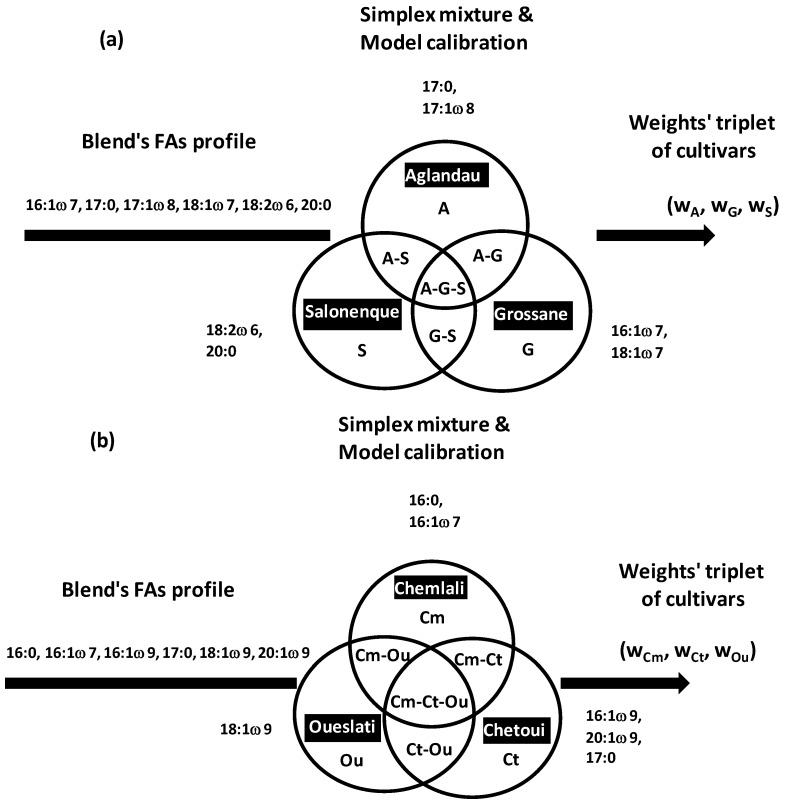
Application of simplex network for prediction of weights’ triplets (*w*_1_, *w*_2_, *w*_3_) giving proportions of three French (**a**) and three Tunisian (**b**) mixed OO cultivars in trivarietal OO blends characterized by fatty acid profiles.

Simplex approach in OO field was recently applied for composition analysis of French and Tunisian trivarietal blends by prediction proportions of the three co-occurring VOO cultivars [14,16]: *Aglandau* (*A*), *Grossane* (*G*), *Salonenque* (*S*) for French VOOs [14], and *Chemlali* (*Cm*), *Chetoui* (*Ct*), *Oueslati* (*Ou*) for Tunisian VOOs [16] (Figure 22a,b). The approach is easily extensible to blends containing more than three groups.

Proportions’ triplets of (*A*, *G*, *S*) and (*Cm*, *Ct*, *Ou*) were predicted from calibration models based on complete sets of *N* = 66 and 231 mixtures, respectively. These numbers resulted from combinations of *q* = 3 VOO components by blocks of *w* = 10 and 20 samples, respectively. Each of the *N* (66 or 231) blends was characterized by a weights’ triplet (*w_A_*, *w_G_*, *w_S_*) or (*w_Cm_*, *w_Ct_*, *w_Ou_*). Proportion of VOO_k_ is given by dividing *w_k_* by the constant sum *w* = Σ*w_k_* = 10 (French blends) or 20 (Tunisian blends). The higher total weight *w* = 20 in Tunisian blends was due to the fact that more contrasted or overlapped variations were initially observed between and within Tunisian VOOs compared to the three French ones. It was shown that increase in w makes to absorb more variability between and within mixed VOOs leading to prediction errors reductions.

At the output of each mixture (*w*_1_, *w*_2_, *w*_3_), an average FA profile was calculated from the w profiles shared between *w*_1_, *w*_2_, *w*_3_ individuals randomly sampled from the 1st, 2nd and 3rd VOO, respectively. For the complete set of *N* = 66 (*A*, *G*, *S*) and 231 (*Cm*, *Ct*, *Ou*) blends, 66 and 231 average FA profiles were calculated as barycentre responses of the three combined VOOs. These two elementary response matrices of average 66 and 231 FA profiles were iterated 30 times to absorb (integrate) chemical variations within and between cultivars. The number of repetition *k* was fixed to 30 because averages of different FAs in different blends stabilized from the 25th iteration. Finally, the 30 × 66 (1880 French) and 30 × 231 (6930 Tunisian) simulated blends were used as extensive background chemical basis to predict the 66 (*A*, *G*, *S*) and 231 (*Cm*, *Ct*, *Ou*) weights’ triplets, respectively. Predictive models were performed by linear discriminant analysis linking the ordinal weights’ triplets (*w*_1_, *w*_2_, *w*_3_) to a set of discriminant FAs. Six most discriminant FAs were used in French and Tunisian models: 16:1ω7, 18:1ω7, 17:0, 17:1ω8, 18:2ω6, 20:0 for French samples and 16:0, 16:1ω7, 16:1ω9, 17:0, 18:1ω9, 20:1ω9 for Tunisian samples. Prediction errors of VOOs’ proportions were ≤10% both for French and Tunisian blends. 

Beyond prediction of cultivars’ proportions in blends, simplex mixture network approach was used to determine minimal errors’ area in simplex space leading to identify blends with potentially high traceability for next RDA or PDO development. Error minimization applied on Tunisian VOOs showed that the most predictable blends were:

- Pure *Cm* among the monovarietal blends.
- 50% *Ct*, 50% *Ou* for bivarietal blends.
- 10% *Cm*, 30%–35% *Ct*, 55%–60% *Ou* for trivarietal blends.

## 5. Conclusions

Olive oils are complex matrices characterized by high chemical variability due to multiple composition influencing factors including:

- Olive living (culture) conditions including varietal types and geographical origins.
- OO preparation ways giving pure, mixed and adulterated samples.
- Variable proportions of different constitutive components of final OO blends.

To better control the effects of these potential factors, OO samples are chemically analysed then statistically treated in order to:

- Qualitatively determine monovarietal (pure), binary (mixed) or multivarietal (blend) aspects.
- Quantitatively evaluate proportions of different mixed components.
- Conclude about the most discriminant or predictive chemical variables in the built qualitative and quantitative models.

Chemical analyses of OO include chromatographical profiles of several metabolic families (fatty acids, phenols, volatiles, etc.) and spectroscopic features containing several hundreds of recorded wavenumbers. Chemometrical analyses of chromatographic and spectroscopic data can be carried out by several methods helping for highlighting specific characteristics (i), authentic classifications (ii) and reliable predictive fingerprints (iii) of different OO samples: 

Specific characteristics associated with differentiation poles of OO samples (i) can be highlighted by topological analysis including PCA and CA. 

Authenticity of different OO samples (ii) can be defined by classification methods based on distance calculation including HCA. 

Fingerprint (traceability) analysis of different co-occurring OO components in blends (iii) can qualitatively performed by means of different recognition patterns’ methods and network-based approaches. Recognition patterns’ methods include LDA, PLS-DA, SIMCA, SVM and KNN. Network approaches cover many algorithms based on ANNs. Under quantitative aspect, traceability of OO blends can be analysed by predicting proportions of several co-occurring components using simplex-approach. In the case of high number of candidate predictive variables (particularly for spectroscopic data), PLS can be used to select the most significant ones.

Finally, results issued from the different chemometrics methods are helpfully used for (i) routine quality control of OO samples with respect to known compositions and origins, (ii) adulteration detection by reference to well-defined quality (consumption) norms, (iii) outlining new labelled commercial products within a statistically surrounded framework (e.g. RDO, PDO), etc.
